# Sociodemographic characteristics of women in a public hospital in Campinas who underwent legal abortion due to sexual violence: cross-sectional study: Características sociodemográficas de mulheres que realizaram aborto legal decorrente de violência sexual em um Hospital Público em Campinas: estudo transversal

**DOI:** 10.1590/1516-3180.2017.0048150317

**Published:** 2017-07-31

**Authors:** Danielle dos Santos Mutta, Daniela Angerame Yela

**Affiliations:** I Undergraduate Student, School of Medical Sciences, Universidade de Campinas (UNICAMP), Campinas (SP), Brazil.; II MD, PhD. Professor, School of Medical Sciences, Universidade de Campinas (Unicamp), Campinas (SP), Brazil.

**Keywords:** Sex offenses, Abortion, legal, Rape

## Abstract

**CONTEXT AND OBJECTIVE:**

Sexual violence is increasingly frequent worldwide. The aim here was to evaluate the sociodemographic and psychological characteristics of women who requested legal abortion, at a public healthcare service, after suffering sexual violence.

**DESIGN AND SETTING:**

Retrospective descriptive study on 131 women who underwent legal abortion at the University of Campinas between 1994 and 2014, consequent to sexual violence.

**METHODS:**

The sociodemographic and psychological characteristics of women who were victims of sexual violence were evaluated from their medical records. The tests used to evaluate possible associations were the chi-square and/or Fisher’s exact test.

**RESULTS:**

The women’s mean age was 23 ± 9.2 years; 77.9% were white and 71.8% were single; 32.8% were students and 58.6% had employment outside of their homes. The majority reported that they did not know the aggressor (62.3%), but among the adolescents, 58% of the aggressors were known. The majority asked for abortion up to the 12^th^ weeks of gestation (63.4%). Only 2.3% presented curettage complications. The psychological situation most frequently encountered was determined, in 34.4% of the cases before the abortion; and good in 32.8% after the abortion.

**CONCLUSIONS:**

There was greater occurrence of sexual violence among students and women who worked outside. Among the students, most of these were adolescents and had no previous sexual life. The teenagers were raped by a known aggressor.

## RESUMO

**CONTEXTO E OBJETIVO:**

A violência sexual está cada vez mais frequente no mundo. O objetivo foi avaliar as características sociodemográficas e psicológicas das mulheres que solicitaram o aborto legal em um serviço público após violência sexual.

**DESENHO DO ESTUDO E LOCAL:**

Estudo descritivo retrospectivo com 131 mulheres que realizaram aborto legal após violência sexual na Universidade Estadual de Campinas no período de 1994 a 2014.

**MÉTODOS:**

Foram avaliadas as características sociodemográficas e psicológicas das mulheres vítimas de violência sexual por meio de seus prontuários. Os testes utilizados para avaliar possíveis associações foram o qui-quadrado e/ou o teste exato de Fisher.

**RESULTADOS:**

A idade média foi de 23 ± 9,2; 77,9% eram brancas e 71,8% solteiras; 32,8% eram estudantes e 58,6% trabalham fora. A maioria relatou desconhecer o agressor (62,3%), porém entre as adolescentes, 58% dos agressores eram conhecidos. A maioria das mulheres solicitou o aborto com até 12 semanas de gestação (63,4%). Apenas 2,3% apresentaram complicações decorrentes da curetagem e a situação psicológica mais encontrada foi decidida em 34,4% no pré-aborto e bem em 32,8% dos casos no pós-aborto.

**CONCLUSÕES:**

Houve maior ocorrência de violência sexual entre estudantes e mulheres que trabalham fora. As estudantes, na maioria, eram adolescentes sem vida sexual prévia. As adolescentes foram violentadas por agressor conhecido.

## INTRODUCTION

In 1994, the Organization of American States (OAS) defined violence against women as “any gender-based act that causes death, injury, or physical, sexual or psychological suffering to women, both in public and private spheres”.^1^ Sexual violence is one of the oldest and bitterest expressions of gender violence and ­violation of human rights, sexual rights and reproductive rights.^2^

Multiple health problems resulting from sexual violence also need to be considered. These may include: physical damage, death and morbidities resulting from sexually transmitted diseases, such as HIV infection. There is also psychological damage that produces intense and devastating effects that are sometimes irreparable.^3^ Pregnancy resulting from sexual violence is a complex process due to emotional, familial, social and biological impacts. This unwanted pregnancy is taken by many women to be a second form of violation and, because it becomes intolerable and impossible to maintain until the end, they resort to abortion.^4^

Abortion in cases of pregnancy due to sexual violence does not depend on judicial authorization in Brazil. A woman who has suffered sexual violence does not have any duty to report the occurrence to the police; nor is she obliged to submit a report to the police or to undergo medical-legal examination. However, she should be supported in taking appropriate police and judicial measures. Failure to take such action does not constitute a legal basis for denying abortion.^5^^,^^6^

## OBJECTIVE

This study aimed to report on the physical and psychological situation of women who sought care through the Brazilian National Health System after having suffered sexual violence and become pregnant; and to characterize the care provided at a university reference service in the state of São Paulo for the practice of legal abortion.

## METHODS

### Design, setting and ethics

This was a retrospective descriptive study on women who underwent abortion induced due to sex violence. The abortion procedures were carried out at the University of Campinas (Universidade de Campinas, Unicamp) between January 1994 and December 2014. This study was approved by our institution’s Research Ethics Committee, under number 4370353/1.

### Participants

The sample size was obtained according to convenience, i.e. it comprised all women who requested abortion due to sexual violence during the study period.

### Variables and statistical analysis

The variables analyzed were as follows: age (classified in three categories: up to 20 years, 21-30 years or more than 30 years), color (white, black or brown), marital status (single, married or others), religion (catholic, evangelical, spiritist or atheist), schooling (illiterate, completed elementary school or incomplete high school), monthly income (up to 5 minimum wages or more than 5 minimum wages), profession [student, employee (working outside of home) or housewife], sex life (yes or no), gestation (yes or no), parity (nulliparous or multiparous), cesarean section (yes or no), abortion (yes or no), weight (in kilograms), height (in meters), body mass index (BMI categorized as low weight < 20 kg/m^2^, adequate weight 20-25 kg/m^2^, overweight 25-30 kg/m^2^ or obese > 30 kg/m^2^), aggressor type (known or unknown), number of abusers (one, two or three), intimidation used (verbal, physical aggression or use of knives or firearm), type of relationship (vaginal or vaginal and others), injuries, police report (yes or no), associated diseases (other diseases presented by the women, such as diabetes mellitus, hypertension, heart diseases, thyroid diseases and others), gestational age (up to 12 weeks or over 12 weeks), use of emergency contraception (yes or no), length of hospital stay (in days), amount of misoprostol (number of tablets used), oxytocin use (yes or no), curettage (yes or no), post-abortion complications (yes or no) and psychological status before and after the abortion. All variables were obtained from the medical records. The psychological status was obtained through the description provided by the psychologist, who, at the end of his analysis, concluded with and described the psychological status of the woman in the medical record. This status was registered as “determined” (i.e. the woman had no psychological conflict with the decision) or “not determined” (i.e. the woman wanted the pregnancy to stop but considered that the situation was embarrassing).

The frequencies, means and standard deviations of the sociodemographic characteristics of these women were calculated. The tests used to evaluate possible associations were the chi-square and/or Fisher’s exact test. SAS 9.4 was used to perform these procedures.

## RESULTS

During the study period, 131 victims of sexual abuse sought termination of pregnancy at our institution. The mean age of the women was 23 ± 9.2 years, and evaluation according to age group showed that 36.6% of the women were between 10 and 20 years of age, 35.9% between 21 and 30 years and 27.5% between 31 and 41 years. Among these women, 77.6% were white, 16% brown and 6.1% black. Regarding marital status, 71.8% were single, 10.7% were married, approximately 10% were divorced and 7.3% had other marital status. Regarding religion, 48.8% were Catholic, 32.5% evangelical, 3.2% spiritist, 15.7% had no religion and for 6.1% there was no information about religion in their medical record (Table 1).

**Table 1.**
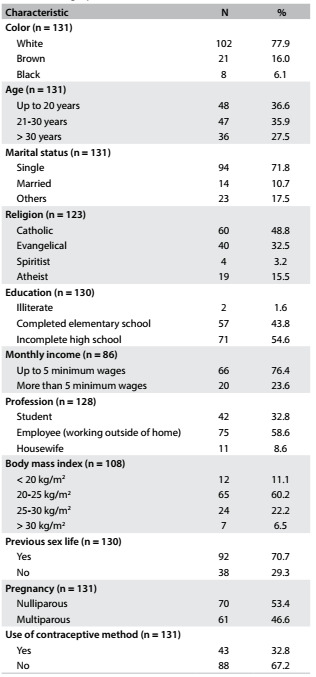
Percentage distribution of women who requested termination of pregnancy due to sexual violence, according to sociodemographic characteristics and antecedents

Considering monthly income, 76.4% of the women had a low income of up to five monthly minimum wages; regarding schooling level, 45.4% of them had attended elementary school. Regarding occupation, 32.8% were students and 58.6% had employment outside of their homes. Regarding sex life before the rape, 29.3% of the women had not started their sex life, 53.4% were nulliparous and 67.2% were not using any contraceptive method (Table 1).

Most of the women reported that they did not know the aggressor (62.3%). However, in the adolescent age group, 58.3% of the aggressors were known. Most of the acts of violence were perpetrated by one aggressor (93.5%). The women had more frequently been intimidated through physical aggression and verbal threat (54.9%) and only a few through personal injuries at the time of the consultation (3.1%) (Table 2).

**Table 2.**
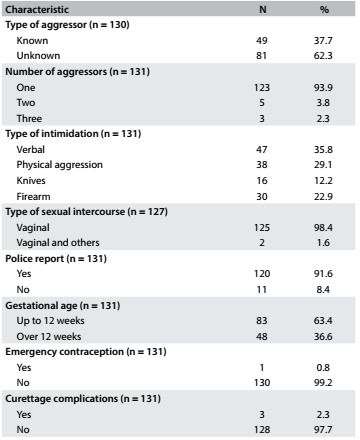
Percentage distribution of women who requested termination of pregnancy due to sexual violence according to characteristics of violence and termination of pregnancy

Although presentation of a police report at the hospital was not mandatory, 91.6% of the women who underwent legal abortion presented this document. Chronic comorbidities, such as diabetes mellitus, systemic arterial hypertension and thyroid disease were not present in any of the women, possibly because they were young. However, 12.2% reported smoking.

Among the women who requested abortion, 63.4% were not more than 12 weeks pregnant and 36.6% were between 12 and 20 weeks pregnant. Emergency contraception was not used by 99.2%. To induce abortion, 56.5% of the women received up to 20 misoprostol tablets, and 3.8% used oxytocin in association with this.

Only 2.3% of the women presented complications (two cases of cervical lacerations and one of uterine perforation) due to curettage, which all the women underwent. Regarding psychological state, 34.4% of the women stated that they were committed to having a legal abortion at the time of the pre-abortion consultation and 32.8% declared themselves to be well at the time of the abortion. All the women were determined to stop the pregnancy. Of these, 34.4% did not have any psychological conflicts with the decision and therefore they were considered to be “determined” and the rest wanted to stop the pregnancy, but considered that the situation was embarrassing.

In comparing the study variables between the age groups, which were established as up to 20 years of age for adolescents and 21 years of age and over for adults, we found significant differences relating to type of aggressor, previous sex life, use of contraceptive method and occupation (Table 3).

**Table 3.**
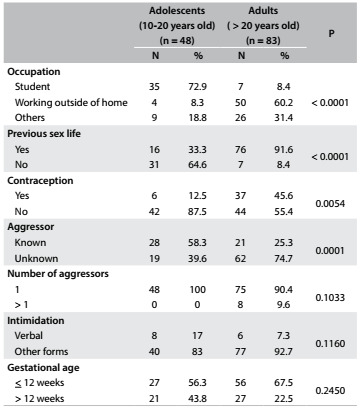
Characteristics of women and sexual violence according to age

## DISCUSSION

At our service, over the last 20 years, 131 women discontinued their pregnancies due to sexual violence. In a service in Rio de Janeiro, 156 women terminated their pregnancies over a three-year period. In the Federal District, 21 women stopped their pregnancies between 1996 and 2001.^7^

The characteristics of the women in the present study who experienced violence (mostly young white single childless women) were similar to those in other studies in the literature.^8^^,^^9^^,^^10^^,^^11^

In this study, the aggressor was unknown in 62.3% of the cases. However, among the adolescents, 58% of the cases of violence were committed by known aggressors. In a hospital in São Paulo, the perpetrator was unknown in most cases of violence against women, both among adolescents and among adults. Regarding the numbers of aggressors and the types of violence, that study and the present study had similar results, with predominance of violence by a single aggressor and vaginal sex as the type of violence.^12^

At our service, almost 92% of the women presented a police report, although this was not necessary for terminating a pregnancy due to sexual violence. However, most services choose to require a police report and often also a report from the Medical Legal Institute (MLI) to prove that the woman was actually raped (given that in Brazil, abortion is legal only under certain circumstances). In other words, it is assumed that women might lie and that professionals would need a guarantee that they were not being deceived, in order to provide this form of care. This is an attitude that reveals non-recognition of women as autonomous and responsible beings by professionals. It constitutes an important obstacle against caring for rape victims, who often fail to seek healthcare because they feel constrained by the requirement to firstly go and make report at a police body.^13^

In comparing the study variables between the age groups, i.e. from 10 to 20 years (taken to be adolescents) and from 21 to 41 years (taken to be adults), we found significant differences in relation to type of aggressor, previous sex life, use of contraceptive method and profession. Regarding the type of aggressor, 58% of the adolescents reported that the aggressors were known, while 75% of the adults reported that they were unknown. We believe that this difference between the types of aggressor (known or unknown) relates to the fact that adolescents are more susceptible to this type of violence, especially at the hands of aggressors who are part of their routine and exercise more control over maintaining their silence.

Regarding previous sex life, 65% of the adolescents had not had any sex life prior to the sexual violence, while 92% of adults had had sex. This difference was expected, since the group of adolescents included some very young women. We believe that the psychological impact on these young women is greater, since their first sexual experience was through violence. A study on 117 women showed that 33% of them had not started to have a sex life, and that 28.7% were aged 10-19 years.^10^ According to the literature, this is a probable an aggravating factor regarding these women’s sexuality, especially when this violence results in pregnancy.^14^

Regarding use of contraceptives, 87.5% of the adolescents were not using them, while 55% of the adults also were not using them. The non-use of prior contraception by these adolescents may be explained by the fact that many had not had any previous sex life. Nonetheless, more than half of the adult women also were not using contraception. Regarding occupation, 73% of the adolescents were students and 60% of the adults had employment outside of their homes, which possibly made them more exposed to sexual violence.

One limitation of this study was the fact that these data were collected retrospectively on the basis of annotations in the subjects’ medical files by different professionals and much information was unavailable due to incompleteness of the records.

A multicenter study conducted in Brazil, in which 4631 young adults aged 18-24 years were interviewed, showed that 21% of the women reported having had at least one episode of induced abortion and that 23% of the women’s sexual intercourse resulted from coercion.^15^ This study showed that there was a situation of vulnerability among young adult women, and that violence against these women was going unreported, thus resulting in life-threatening situations such as unsafe abortion. Women’s lack of knowledge of their right to terminate pregnancy in the event that this resulted from rape ultimately violates women’s sexual rights. Women sometimes only become aware of such rights in seeking healthcare services consequent to discovering their gestational state.^16^

Brazilian healthcare professionals’ attitudes also contribute towards violation of these rights. They may lack training, information and knowledge about legal abortion procedures that are provided by the healthcare system. They may also fear being deceived by rape victims, whose reports of pregnancy due to rape are questioned, either for moral or religious reasons.^16^^,^^17^^,^^18^

Thus, we hope that these data will contribute towards expansion of policies that may provide more information to women and health professionals about legal abortion in order to try to reduce the damage suffered by these women.

## CONCLUSION

There was greater occurrence of sexual violence among students and women who worked outside. Among the students, most of these were adolescents and had no previous sexual life. The teenagers were raped by a known aggressor.
